# Comparative transcriptomic analysis of pyrethroid-resistant Anopheles gambiae s.l. from Ghana, reveals concentration-dependent and site-specific patterns of gene expression

**DOI:** 10.21203/rs.3.rs-7760720/v1

**Published:** 2025-12-16

**Authors:** Christopher Mfum Owusu-Asenso, Anisa Abdulai, Isaac Kwame Sraku, Simon Kwaku Attah, Fred Aboagye-Antwi, Yaw Asare Afrane

**Affiliations:** University of Ghana Medical School; University of Ghana Medical School; University of Ghana Medical School; University of Ghana Medical School; University of Ghana; University of Ghana Medical School

**Keywords:** Pyrethroid resistance intensity, metabolic gene expression, concentration-dependent gene expression, site-specific gene expression, Anopheles gambiae s.l., kdr mutations

## Abstract

The role of detoxification enzymes in pyrethroid resistance intensity among malaria vectors remains a critical area of research. This study evaluated the role of detoxifying enzymes in driving resistance intensity in *Anopheles* mosquitoes from high insecticide resistance sites in Ghana. Larvae were collected from Tema, Abossey Okai, and Dansoman, and bioassays were performed on 3–5 days old adult females by exposing them to deltamethrin at discriminating concentrations (1× = 0.05%, 5× = 0.25%, and 10× = 0.5%) to assess resistance intensity. A piperonyl butoxide (PBO) synergist assay was used to test the involvement of *cytochrome P450s*, while qRT-PCR quantified expression of detoxification genes (*CYP6P1, CYP9K1, CYP6M2, CYP6P3, CYP4G16, GSTE2*, and *CYP6Z1*). *kdr* mutations (*L995F*, *L995S*) were genotyped. High-intensity resistance was observed across all sites [deltamethrin 10× MR = 75–91%]. Pre-exposure to PBO significantly increased mortality (Tema: 13–56%; Abossey Okai: 20–91%; Dansoman: 34–88%, P < 0.001), however, complete susceptibility was not restored. The *L995F* mutation was present at similar frequencies in resistant and susceptible mosquitoes. Transcriptomic profiling revealed concentration-dependent and site-specific expression: Tema; *CYP9K1, CYP6M2, CYP6P1*, and *CYP6P3* were significantly overexpressed (FC = 43.71–1222.98, *P* < 0.05), while *CYP4G16* expression increased with insecticide concentration. In Abossey Okai, *CYP9K1, CYP6P1, CYP6M2*, and *CYP6P3* were overexpressed (FC = 5.54–162.84). Mosquitoes from Dansoman showed generally low expression, however, *CYP6M2* and *CYP6P3* were overexpressed (FC = 120.80–292.68). These findings may suggest the dominant role of metabolic resistance, particularly *P450*-mediated detoxification in driving high pyrethroid resistance intensity in Ghana.

## Background

The widespread emergence of pyrethroid resistance in malaria vectors poses a critical challenge to global malaria control initiatives, particularly in Ghana, where insecticide-treated nets (ITNs) and indoor residual spraying (IRS) are heavily reliant on this class of insecticides^1^. The upsurge of pyrethroid resistance among *Anopheles* mosquito populations has severely undermined the effectiveness of these interventions^2^, contributing to ongoing malaria transmission even in areas with high coverage of these vector control tools.

The observed resistance pattern is likely influenced not only by widespread *kdr* mutations which alter the voltage-gated sodium channels targeted by pyrethroids, but could also be due to the overexpression of detoxification enzymes such as *cytochrome P450 monooxygenases, glutathione S transferases* (*GSTs*), and *carboxylesterases*^3^. These enzymes play a pivotal role in enhancing the mosquitoes’ ability to metabolize and neutralize insecticides, thereby increasing their survival rate despite insecticide exposure^4^. The *kdr* mutations are prevalent across malaria-endemic areas, and in some regions, have reached fixation in mosquito populations^5^. The coexistence of *kdr* mutations and elevated detoxification enzyme activity may create a polygenic resistance profile threatening malaria elimination efforts^6, 7^.

While *kdr* mutations are well-documented and widely studied, the contribution of detoxification enzymes to pyrethroid resistance intensity, particularly in areas with already high *kdr* frequencies, remains less understood. This knowledge gap is critical, as detoxification enzymes may enhance resistance and influence the effectiveness of insecticides that are increasingly used for vector control. Given the critical role that detoxification enzymes may play in sustaining and intensifying pyrethroid resistance, it is imperative to investigate their contribution to driving resistance intensity in areas where *kdr* mutations are prevalent and possibly fixed. This study aimed to investigate how detoxifying enzymes drive pyrethroid resistance intensity in mosquito populations in areas with high insecticide resistance. Understanding these molecular pathways is crucial, providing insights into how detoxifying genes may heighten resistance intensity in malaria vectors, and also help develop potential interventions that could disrupt these resistance mechanisms and restore the efficacy of current control strategies.

## Results

### Insecticide Resistance Intensity Bioassays and Synergist Bioassays on An. gambiae s.l.

High-intensity resistance was observed in *Anopheles* mosquitoes across all sites; Tema [1x Mortality rate (MR) = 13%, 5x = 81%, 10x = 90%], Abossey Okai [1x MR = 20%, 5x = 34%, 10x = 75%] and Dansoman [1x MR = 34%, 5x = 66%, 10x = 91%]. Exposure of the Kisumu susceptible strain to a standard baseline insecticide concentration [deltamethrin (1x MR = 0.05%)] resulted in full mortality (100%), confirming the susceptibility of this population to the insecticide and effectiveness of the insecticide-impregnated papers, Fig. 2a.

Pre-exposure to PBO (4%) for 1 hour before deltamethrin (0.05%) significantly increased mortality in An. gambiae s.l. populations from Tema (13% to 56%), Abossey Okai (20% to 91%), and Dansoman (34% to 88%) (χ^2^ = 40, df = 1, *P* < 0.001), although full susceptibility was not restored (Fig. 2b).

### Concentration-Dependent Gene Expression Dynamics in Anopheles Mosquitoes

Transcriptomic analysis revealed concentration-dependent metabolic gene expression in deltamethrin-resistant mosquitoes at 1x, 5x, and 10x discrimination doses, with variable gene responses across different insecticide concentrations, Table 1.

In Tema, *CYP9K1, CYP6M2, CYP6P1* and *CYP6P3* were significantly overexpressed in 1x and 10x insecticide concentrations [fold change (FC) = 43.71–1222.98] in deltamethrin-resistant mosquitoes in comparison to the Kisumu susceptible laboratory strain (P < 0.05). Expression levels of *CYP4G16* increased with insecticide concentration [FC: 1x = 11.5, 5x = 15.9, 10x = 26.3], whilst *GSTe2* and *CYP6Z1* were moderately expressed 1x and 10x insecticide concentrations (FC = 1.85–4.20). In Abossey Okai, *CYP9K1*, *CYP6P1* were overexpressed in 1x and 10x concentration [FC = 5.54–162.84] in deltamethrin-resistant mosquitoes, while *GSTe2* [FC = 0.01–2.52], CYP6M2 [FC = 0.07–9.71], *CYP6P3* [FC = 5.54–41.38] and *CYP4G16* [FC = 0.01–0.22] expression remained consistently low across all insecticide concentrations. In Dansoman, the control site; *CYP6M2* and *CYP6P3* were overexpressed in 1x and 10x deltamethrin-resistant mosquitoes (FC = 120.80–292.68), whilst low expression of *GSTe2, CYP9K1, CYP6P1, CYP6Z1* and *CYP4G16* (FC = 0.00 to 1.43) were observed across all insecticide concentrations, Table 1.

### Site-specific Variations in Gene Expression Profiles in Anopheles mosquitoes

Gene expression profiles varied significantly across the three study sites; Tema, Abossey Okai, and Dansoman, indicating site-specific differences in metabolic resistance mechanisms. In Tema, metabolic genes such as *CYP9K1* [FC = 13.11–1222.98] and *CYP6M2* [FC = 6.05–832.25] were highly overexpressed in deltamethrin-resistant *An. gambiae* s.l. compared to that of Abossey Okai and Dansoman, Fig. 3. Comparatively, *CYP6P1* [FC = 10.75–108.20], *CYP6P3* [FC = 32.51–937.41] and *GSTe2* [FC = 0.24–4.20] were also relatively highly expressed in Tema compared to the other study sites, Fig. 3.

Gene expression in *An. gambiae* s.l. from Abossey Okai, was moderate to relatively high, with *CYP9K1* overexpressed [FC = 0.23–162.84] as compared to lower expression levels observed in Dansoman. *GSTe2* [FC = 0.01–2.52], *CYP6M2* [FC = 0.07–9.71] and *CYP6P3* [FC = 5.54–41.38] expression remained consistently low across all insecticide concentrations, Fig. 3. *Anopheles gambiae* s.l. from Dansoman exhibited the lowest overall gene expression levels, with minimal expression of *GSTe2* [FC = 0.03–0.09], *CYP6Z1* [FC = 0.04–0.16] and *CYP9K1* [FC = 1.07–1.43]. However, *CYP6M2* [FC = 25.75–292.68] was overexpressed, Fig. 3.

### Gene Expression in Deltamethrin-Resistant and Susceptible An. gambiae s.l.

To understand the molecular basis of resistance intensity, gene expression between two biologically distinct groups: mosquitoes that died at the diagnostic dose (1×, representing the susceptible phenotype), and those that survived the highest dose (10×, representing the resistant phenotype) were analysed. Comparative gene expression levels between 10x deltamethrin-resistant *An. gambiae* s.l. in comparison to 1x deltamethrin-susceptible *An. gambiae* s.l. indicated substantial overexpression of specific detoxification genes. *CYP6P1* [FC = 43.94] was overexpressed in 10x deltamethrin-resistant *An. gambiae* s.l.in comparison to the 1x deltamethrin-susceptible *An. gambiae* s.l. [FC = 1.98]. Moreover, *CYP9K1* was significantly overexpressed in 10x deltamethrin-resistant *An. gambiae* s.l. [FC C.l. mbiae*P* < 0.001]. Other genes, including *CYP6P3* [resistant: FC = 35.92 vs susceptible: FC = 2.59; *P* = 0.002] and *CYP4G16* [resistant: FC = 26.33 vs susceptible: FC = 0.13; *P* = 0.002] were significantly overexpressed in 10x deltamethrin-resistant *An. gambiae* s.l. in comparison to 1x deltamethrin-susceptible *An. gambiae* s.l., Table 2.

### kdr Mutation in deltamethrin-resistant and susceptible Anopheles gambiae s.l.

A total of 177 *An. gambiae* s.l. were genotyped for the presence of *kdr* mutations. A high allele frequency (0.78) of *L995F* was observed in Tema. In contrast, the *L995S* mutation was present at a much lower frequency in Tema (0.12) (*χ*2 = 57, *P* < 0.001). In Abossey Okai, significantly high allele frequency (0.72) of *L995F* were observed (*χ*^2^ = 60, *P* < 0.001). However, low allele frequency of 0.03 was recorded for the *L995S* mutation in Abossey (*χ*^2^ = 60, *P* < 0.001).

The *L995F* mutation had a relatively low frequency (0.41) in Dansoman compared to Tema and Abossey Okai. Similarly, the *L995S* mutation frequency in Dansoman was low (0.19), Table 3.

### Species Discrimination in Anopheles gambiae s.l.

A total of 177 *An. gambiae* s.l. specimens were analysed to differentiate between the sibling species. Overall, *An. coluzzii* was the dominant species 88.1% (156), followed by *An. gambiae* s.s. 8.5% (15) and then hybrids 3.4% (6). Site-specific data revealed that, *An. coluzzii* was the most dominant species in all study sites. Hybrids were only present in Dansoman, Fig. 4.

## Discussion

Understanding insecticide resistance mechanisms in *Anopheles* mosquito populations is essential for effectively combating malaria transmission in areas with high insecticide resistance. This study highlights the significant role of detoxification enzymes in insecticide resistance intensity. The overexpression of key metabolic genes may signify an adaptive response of these malaria vectors under increased insecticide pressure and environmental contaminants, indicating the significant role of metabolic resistance mechanisms in driving insecticide resistance intensity in *Anopheles* mosquitoes.

The observed high-intensity resistance to deltamethrin indicates the possibility of strong selection pressure within these sites. A similar trend was observed in studies that reported significant pyrethroid resistance in *An. gambiae* populations from Ghana^8^, Nigeria^9^ and Burkina Faso^10^. These findings may suggest a challenge in controlling these vectors using pyrethroid-based interventions. Pre-exposure to PBO bioassays significantly increased deltamethrin-induced mortality in malaria vectors from all study sites, suggesting that monooxygenases may play a major role in the observed pyrethroid resistance in this study^11^. However, full susceptibility was not restored. This observation may indicate that other resistance mechanisms or genes, beyond *monooxygenases* such as cuticular resistance genes, may be contributing to resistance in the mosquitoes. This is particular concerning, indicating that the recently introduced PBO-bednets distributed by the National Malaria Elimination Program may have no operational effect on the control of *An. gambiae* s.l..

The concentration-dependent overexpression of *CYP4G16* in Tema, with fold-change values significantly increasing systematically from 1x to 10x, could suggest that this gene actively contributes to detoxification under increasing pyrethroid insecticide. *CYP4G16* is associated with cuticular resistance mechanisms in *Anopheles* species, where its elevated expression improves cuticle impermeability to insecticides^12^. This systematic increase in *CYP4G16* expression aligns gene expression patterns from other studies where increasing deltamethrin concentrations resulted in overexpression of detoxifying genes^11, 13^. However, *CYP4G16* expression was negligible in Abossey Okai and Dansoman, which may indicate its site-specific role, potentially due to the heightened xenobiotics in Tema^14^. These site-specific variations may reflect the effect of localized selection pressures influencing detoxification gene regulation in *An. gambiae* s.l. populations.

The *CYP6M2* and *CYP9K1* genes showed significant overexpression in the Anopheles population from Tema indicating their possible role in pyrethroid detoxification. This elevated expression contrasts with much lower levels observed in Abossey Okai, indicating a possible variation in selective pressures between the sites. Other studies have reported similar variations in gene expression patterns from sites with different environmental pressures^14, 15^.

High gene expression levels in deltamethrin-resistant *An. gambiae* s.l. exposed to 1x and 10x insecticide concentration followed by a sharp decrease in gene expression levels in *Anopheles* mosquito samples exposed at 5x insecticide concentration may suggest a complex regulatory mechanism that may depend on insecticide concentration^13^. This expression pattern could imply a possible saturation of detoxification at 5x, followed by adaptive upregulation at 10x to cope with higher insecticide exposure^13^. This finding may suggest that reducing insecticide use could help manage resistance by lowering selective pressure.

Interestingly, 1x deltamethrin-susceptible *An. gambiae* s.l., despite harbouring *kdr* mutations relatively similar to the 10x deltamethrin-resistant *An. gambiae* s.l., showed lower expression of these detoxifying genes. The low expression of detoxifying genes in the 1x deltamethrin-susceptible *An. gambiae* s.l., may have contributed to their higher mortality despite the presence of these *kdr* mutations. This finding suggests that detoxifying enzymes may play a more significant role in high-pyrethroid resistance intensity than *kdr* mutations, as metabolic resistance mechanisms may be the primary factor driving survival under pyrethroid exposure. These results are consistent with previous studies that reported on the importance of detoxification pathways, such as *cytochrome P450s*, in insecticide resistance^11, 13^. However, *GSTE2* and *CYP6Z1* showed minimal variation in expression (DELTA 1x S vs DELTA 10x R), suggesting that they may play a lesser role in pyrethroid resistance, supporting previous studies that reported variable contributions of different detoxification enzymes to insecticide resistance^10 15^.

The *L995F* mutation was observed at high frequencies across all study sites. These findings are consistent with other studies documenting the widespread presence of the *L995F* mutation in *Anopheles* populations exposed to intense pyrethroid use^16, 17, 18^. Furthermore, the polygenic nature of insecticide resistance, as indicated by the co-occurrence of *kdr* mutations and elevated expression of detoxification genes could pose a significant challenge to malaria control efforts in these localities.

Species composition analysis revealed that *An. coluzzii* was the predominant species of the sampled *Anopheles* population. This aligns with previous findings that have reported that *An. coluzzii* has adapted well to urbanized and polluted environments, likely due to its ecological flexibility^19, 20^.

## Conclusion

This study revealed critical knowledge on the insecticide resistance mechanisms in *An. gambiae* s.l. population in Ghana, indicating the concentration-specific and site-specific roles of detoxification enzymes in high pyrethroid-resistance intensity. Overexpression of detoxifying genes was significantly associated with high pyrethroid-resistance intensity in *Anopheles gambiae* mosquitoes. Allele frequency of *kdr* mutation was relatively similar among resistant and susceptible *Anopheles* mosquitoes. These findings may suggest the dominant role of metabolic resistance in driving high-pyrethroid resistance intensity in *An. gambiae* s.l. in Ghana.

## Materials and Methods

### Study Design

**Figure 1:**
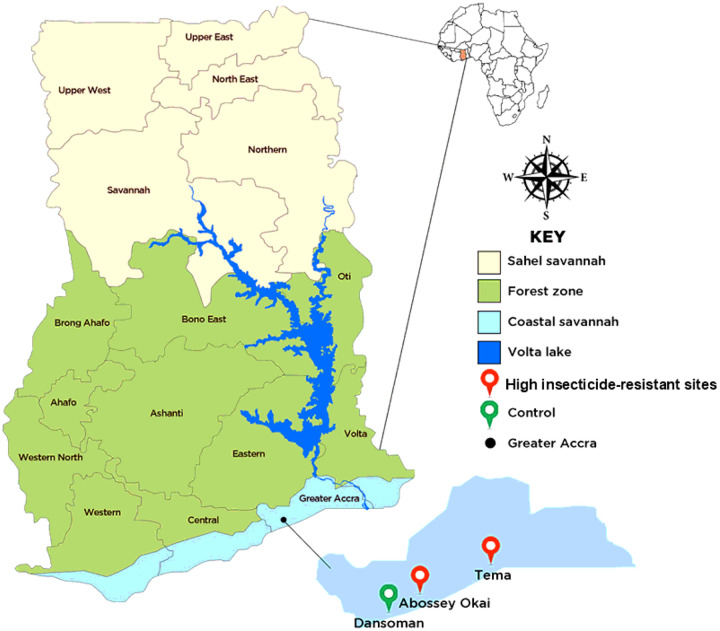
A map of Ghana showing the study sites. (The base map for the study site depiction was sourced from https://ghana-mission.co.in/mapofghana/ and modified using Adobe Photoshop (Version 7.0.1).)

**Figure 2:**
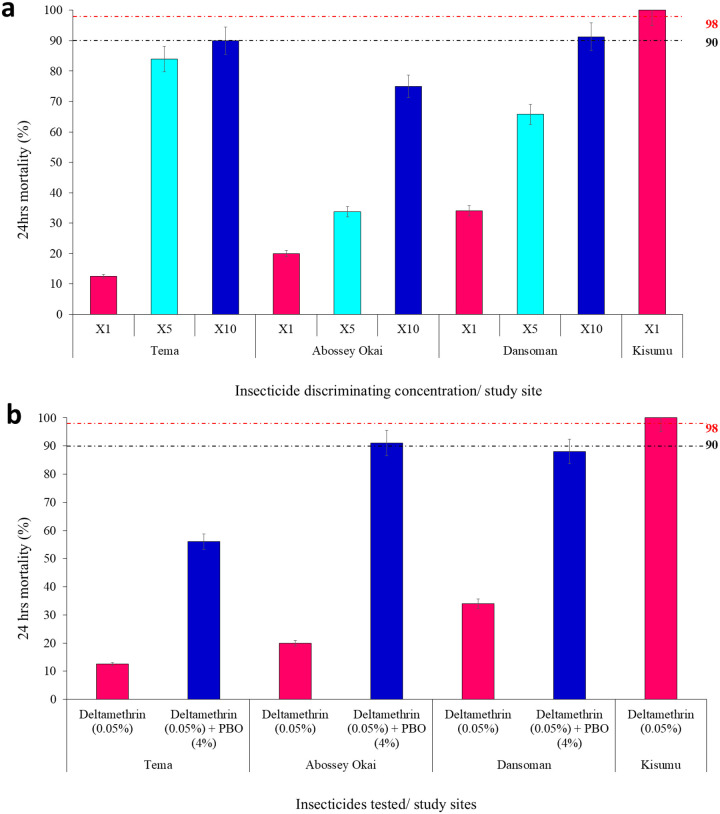
WHO susceptibility bioassays, a: intensity Bioassays b: PBO bioassays on *Anopheles* Mosquitoes from Tema, Abossey Okai, Dansoman and Kisumu Susceptible Strain. Error bars indicate 95% confidence intervals, (R): resistant or alive mosquitoes.

**Figure 3:**
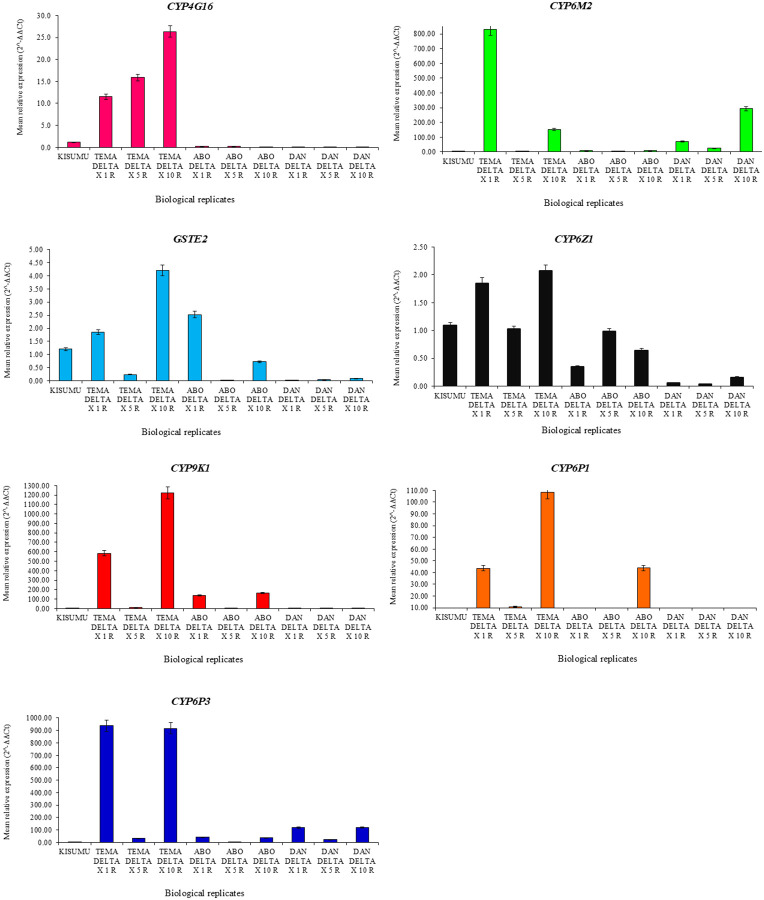
Relative gene expression (*CYP4G16, CYP6M2, GSTe2, CYP6Z1 and CYP9K1, CYP6P1, CYP6P3*) for deltamethrin-resistant *An. gambiae* s.l. populations from Tema, Abossey Okai, Dansoman and Kisumu susceptible strain. Error bars indicate 95% confidence intervals, (R): resistant or alive mosquitoes.

**Figure 4:**
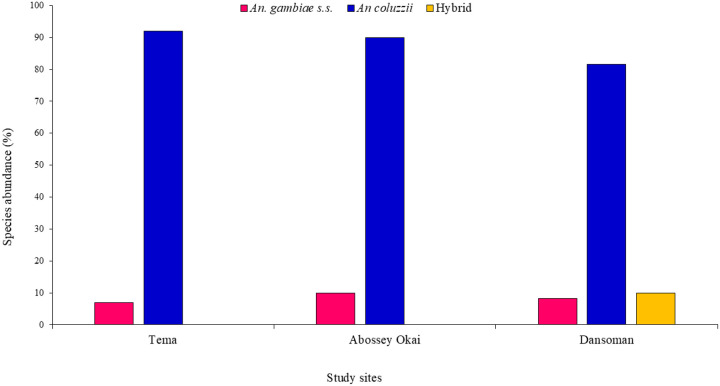
Species Discrimination of *An. gambiae* s.l. from Tema Abossey Okai and Dansoman.

This study was conducted in three sites within Ghana’s coastal savannah ecozone: Tema (5.6698° N, 0.0200° E), Abossey Okai (09°24.886 N, 000°50.939 E), and Dansoman (5° 33′ 0″ N, 0° 16′ 0″ W), Fig. 1.

Tema Community 1 (5.6698° N, 0.0200° W), located in the coastal savannah of southern Ghana, is an industrial and port city. The dense concentration of manufacturing facilities and port operations generates significant industrial effluents, many of which are discharged into open drains and stagnant pools that double as mosquito breeding grounds. The Tema port experiences a constant influx of goods, vehicles, and people from other countries, potentially facilitating the introduction of mosquito populations with diverse genetic backgrounds, including those carrying resistance traits.

Abossey Okai (5.5480° N, 0.2424° W), located in the city of Accra, in the coastal savannah zone of southern Ghana. Oil spills from engine oil changes and vehicle repairs, and the leaching of metal compounds into mosquito breeding habitats, may trigger an adaptive response and increase resistance in the vectors. These contaminants may activate detoxification enzyme pathways such as cytochrome P450s in mosquitoes, helping them metabolize toxins and insecticides, and contributing to enhanced resistance in mosquito populations.

Dansoman, a suburb of Accra, was selected as a control site due to its limited exposure to industrial activities and automobile contamination by oil-spills. This site provides a baseline for comparison with the high-resistance sites in Tema and Abossey Okai.

### Larval Collection and Raising in Insectary

*Anopheles* larvae sampling was carried out from January 2023 to July 2024. To avoid the collection of sibling species, larvae were sampled randomly from different breeding habitats in each study site. The collected larvae were carefully transferred into sterile plastic containers and promptly transported to the insectary at the Department of Medical Microbiology, University of Ghana Medical School. In the insectary, larvae were fed with Tetramin Baby Fish meal and reared under controlled, standardized temperature (26 ± 2°C) and relative humidity (80% ± 10%) with 12 h: 12 h light/dark cycle. Upon pupation, pupae were collected, transferred to cages, and allowed to emerge as adults. From the day of emergence, adults were provided with a wad of cotton wool soaked with 10% sugar solution until ready to be used for bioassay tests.

### WHO Intensity Bioassay on Adult Anopheles mosquitoes

To determine the intensity of resistance in the *An. gambiae* s.l. population, batches of 25 non-blood-fed female mosquitoes aged 3–5 days were subjected to the WHO susceptibility test bioassay. For each insecticide concentration, four replicates and two control tubes were used. Mosquitoes were exposed to papers impregnated with deltamethrin at 1× (0.05%), 5× (0.25%), and 10× (0.5%) concentrations, alongside oil-impregnated papers as controls, following the standard WHO tube assay procedure.^21^. Mosquitoes were exposed for 1 h and the knockdown was recorded every 10 min during the 60-min exposure period. Mortality was recorded after a 24-h recovery period.

Post-bioassay, alive (resistant), dead, and moribund mosquitoes from each insecticide treatment (1x, 5x, 10x) were processed separately. For DNA-based analyses, including molecular species identification and genotyping of *kdr* mutations, the head and thorax of resistant (alive) and dead mosquitoes were individually tweezed using sterile forceps and placed in 1.5 ml Eppendorf tubes containing silica gel and cotton. For RNA-based analyses, both resistant and moribund (susceptible) mosquitoes (mosquitoes unable to stand or fly properly, showing signs of severe incapacitation, such as twitching or lying on their backs) were used. The abdomen, legs, and wings of each moribund and resistant mosquito were submerged in separate 1.5 ml Eppendorf tubes containing RNA later (Ambion), treated according to the manufacturer’s instructions, and stored overnight at 4°C to allow the solution to penetrate the mosquito tissue before transfer to a − 20°C freezer.

### Piperonyl Butoxide (PBO) Synergist Bioassays

To understand the role of metabolic detoxification in pyrethroid resistance, piperonyl butoxide (PBO), a synergist that inhibits the specific activity of P450 monooxygenases in insects was used in the resistance bioassay. Each test had four replicates of 25 unfed female *Anopheles* mosquitoes aged 3–5 days were pre-exposed to 4% PBO-impregnated test papers for 1 hr, and then immediately exposed to 0.05% deltamethrin for another hour. One batch of 25 females were exposed to 4% PBO without insecticide and another batch of 25 females were also exposed to deltamethrin (0.05%) only, these served as controls. The number of mosquitoes knocked down after one hour of exposition to the insecticides were recorded. Mosquitoes were then transferred into holding tubes and supplied with a 10% sugar solution soaked in a wad of cotton. Mortality was scored after the 24-hr recovery period.

### RNA extraction and cDNA synthesis for metabolic resistance determination

To investigate the role of detoxifying genes in resistance, deltamethrin-resistant and susceptible *Anopheles* mosquitoes (1x, 5x, 10x) from each site and Kisumu susceptible strain stored in RNA later at − 20°C were grouped in pools of 10 in 1.5-ml Eppendorf tubes. Total RNA was extracted from each pool (deltamethrin-resistant, deltamethrin-susceptible mosquitoes, as well as the Kisumu susceptible strain) using the ZYMO *Quick-RNA*^™^ *Miniprep Kit* following the manufacturer’s protocol. The cDNA was synthesized from 1 μg of total RNA of three biological replicates each [1x, 5x, 10x deltamethrin-resistant and 1x, 5x, 10x susceptible mosquitoes and the Kisumu susceptible strain using Protoscript III (Invitrogen) cDNA synthesis kit with oligodT20 and RNase H, (Invitrogen, New England Biolabs - United Kingdom) according to the manufacturer’s protocol. The total RNA and synthesized cDNA were stored at − 80°C.

### Expression profile of detoxifying genes in Deltamethrin-resistant An. gambiae s.l.

The level of expression of seven resistance-associated genes was validated by qRT-PCR. These include the *GSTe2, CYP6P3, CYP6M2, CYP9K1, CYP9Z1, CYP64G16, CYP6P1*. Reactions were carried out in a final volume of 10μl consisting of 5μl SYBR Green Master Mix (Roche, Indianapolis, IN), 10 μM of each primer and 2.0μl of cDNA. The qRT-PCR assay was performed on the Bio-Rad Opus 96 PCR System (Bio-Rad) with an initial denaturation at 95°C for 10 min, followed by 40 cycles of 95°C for 10s, 60°C for 10s). Standard curves for all primer sets were carried out using Kisumu cDNA as a reference.

### kdr Genotyping and Species Discrimination of Anopheles Mosquitoes

*kdr* genotyping was done by extracting DNA using the alcohol precipitation method^22^ from the head and thorax of deltamethrin-resistant and susceptible individual mosquitoes preserved on silica gel. The *L995F* and *L995S* kdr mutations were identified using AS-PCR^23^. Mosquitoes collected were morphologically identified using identification keys by Gillies and Coetzee^24^. Members of the *An. gambiae* s.l. were further identified to distinguish sibling species, with a leg of each mosquito as DNA template using protocols of rDNA PCR by Scott *et al*.^25^ and PCR-RFLP by Fanello *et al*.^26^.

### Data Management and Analysis

Descriptive analyses were performed to visualize WHO susceptibility data, resistant allele frequencies, and mosquito species composition in graphs and tables. WHO insecticide susceptibility levels were classified according to the WHO criteria^21^. The chi-square test was utilised to determine differences in resistant alleles among mosquito populations. The allele frequency of resistance gene markers in the vector population from each site was calculated using the Hardy-Weinberg equilibrium (HWE) formula. The oneway ANOVA and Kruskal-Wallis test was employed to compare relative gene expression levels in *An. gambiae* s.l. population. The cycling threshold (CT) values obtained were used in determining the expression levels of the selected genes using the delta CT method^13^. The housekeeping gene Ribosomal Protein S7 (RPS7) (VectorBase: AGAP010592) was used as an internal control. The fold expression of the genes was calculated using the formula, Fold change (FC) = 2 −ΔΔCT with normalization against the ribosomal protein S7. In all analyses, a P-value ≤ 0.05 was considered statistically significant.

## Figures and Tables

**Table 1:** Association Between Resistance Intensity and Mean Relative Gene Expression Levels Across Insecticide Treatment

Study site	Metabolic genes	Kisumu	RI/Relative mean expression Fold change (FC = 2^-ΔΔCt)			P-value (Bonferroni)			ANOVA
			1x	5x	10x	1x/5x	1x/10x	5x/10x	P-value
	*CYP4G16*	1.16	11.5	15.9	26.3	ns	0.036	ns	0.00
	*CYP6M2*	0.73	832.25	6.05	152.41	< 0.001	0.001	ns	0.00
Tema	*GSTE2*	1.20	1.85	0.24	4.20	ns	ns	0.020	0.02
	*CYP9K1*	1.25	583.25	13.11	1222.98	0.005	0.002	< 0.001	0.00
	*CYP6P1*	1.11	43.71	10.75	108.20	ns	0.029	0.002	0.00
	*CYP6P3*	1.17	937.41	32.51	916.52	0.011	ns	0.013	0.00
Abossey Okai	*CYP9K1*	1.25	138.97	0.23	162.84	0.049	0.021	ns	0.01
	*CYP6P1*	1.11	5.64	0.97	43.94	ns	0.001	< 0.001	0.00
Dansoman	*CYP6M2*	0.73	70.78	25.75	292.68	ns	0.016	0.005	0.00
	*CYP6P3*	1.17	118.79	22.47	120.80	0.001	ns	0.001	0.00

RI = resistance intensity, ns = not significant, 1x, 5x, 10x = discriminating concentration of deltamethrin insecticide, Kisumu = Kisumu susceptible strain

**Table 2:** Association Between Mean Expression Levels of Resistant and Susceptible *Anopheles* Mosquitoes

Genes	Mean relative expression level Fold change (FC = 2^-ΔΔCt)			Kruskal Wallis P-value
	Kisumu	DELTA 1x S	DELTA 10x R	
*CYP6P1*	1.11	1.98	43.94	0.001
*CYP4G16*	1.13	0.13	26.33	0.002
*CYP6Z1*	1.09	0.41	0.65	ns
*GSTE2*	1.20	0.09	0.73	ns
*CYP9K1*	1.25	0.85	162.84	< 0.001
*CYP6P3*	1.17	2.59	35.92	0.002
*CYP6M2*	0.73	0.53	9.79	ns

Kisumu: Kisumu susceptible strain, DELTA 1x S: deltamethrin-susceptible An. gambiae s.l. exposed to 1x concentration of deltamethrin, DELTA 10x R: deltamethrin-resistant An. gambiae s.l. exposed to 10x concentration of deltamethrin

**Table 3:** *kdr* Allele Frequency Distribution in *An. gambiae* s.l. Stratified by Study Site, Phenotype and Species.

Study site	Phenotype	N	L995F					L995S				
			RR	RS	SS	F	P-value	RR	RS	SS	F	P-value
Tema	Resistant	27	15	11	1	0.78	0.18	3	0	24	0.12	0.00
	Susceptible	30	18	12	0			4	0	26		
	Total	57	33	23	1			7	0	50		
Abossey Okai	Resistant	30	23	0	7	0.72	0.00	2	0	28	0.03	0.00
	Susceptible	30	20	0	10			0	0	30		
	Total	60	43	0	17			2	0	58		
Dansoman	Resistant	30	3	20	7			0	11	19		
	Susceptible	30	0	23	7	0.41	0.00	0	12	18	0.19	0.07
	Total	60	3	43	14			0	23	37		
**Species**												
*An. gambiae* s.s.	Resistant	7	4	2	1	0.71	0.43	1	1	5	0.25	0.46
	Susceptible	8	4	1	3	0.56	0.03	0	0	8	0.00	-
*An. coluzzii*	Resistant	53	35	11	7	0.76	0.00	4	9	65	0.08	0.00
	Susceptible	78	34	30	14	0.63	0.12	4	11	63	0.07	0.00
Hybrid	Resistant	2	0	2	0	0.50	0.16	0	1	1	0.25	0.64
	Susceptible	4	0	4	0	0.50	0.05	0	1	3	0.13	0.78

N = samples tested, F = Allele frequency, L995F = kdr west, L995S = kdr east, (if P-value < 0.05, not consistent with HWE)

## Data Availability

The datasets generated during and/or analysed during the current study are available from the corresponding author on reasonable request.
